# Portal-Endocrine and Gastric-Exocrine Drainage Technique in Pancreatic Transplantation

**Published:** 2011-05-01

**Authors:** H. Shokouh-Amiri, G. B. Zibari

**Affiliations:** *Department of Surgery, Willis Knighton/LSUHSC Regional Transplant Center, Louisiana State University Health Sciences Center, Shreveport, USA*

**Keywords:** Technique, Gastric-exocrine drainage, Immunosuppression, Acute rejection, Pancreas transplant

## Abstract

Background: Pancreas transplant (PTx) is an established treatment for patients with diabetes mellitus. Diagnosis of rejection has continued to be problematic. In 2007, a new technique of PTx with portal-endocrine and gastric exocrine (P-G) drainage was first performed at our institution. This technique facilitates access to pancreas allograft.

Objective: To report our experience with the first 30 patients who underwent PTx using P-G technique.

Methods: The first 30 patients who underwent PTx between 2007 and 2009 were studied. In these patients, arterial and venous anastomosis was similar to standard portal-enteric (P-E) technique, though contrary to other techniques of enteric drainage, the end of allograft jejunum was anastomosed to the anterior aspect of the stomach.

Results: Donor and recipient demographic data, number of antigen matches and immunosuppressant were collected. All patients achieved euglycemia. 3 patients underwent pancreatectomy: 2 due to vessel thrombosis and 1 due to chronic rejection. 3 patients died—2 with functioning pancreatic and renal allografts. 7 patients with CMV and 4 patients with rejection were diagnosed with endoscopy of allograft duodenum and treated. 1-year patient and graft survival was 94% and 85%, respectively.

Conclusion: This novel technique of PTx has proven to be safe with good patient and allograft survival. Access to donor duodenum and pancreas allograft via endoscopy is unique to this technique and provides the added advantage of life-long easy access to allograft.

## INTRODUCTION

Transplantation of the kidney/pancreas is a well accepted treatment modality for patients suffering from end-stage renal disease (ESRD) and insulin-dependent diabetes mellitus (IDDM) that reliably results in dialysis free and insulin independent state with euglycemia and normal glycosylated hemoglobin levels. Various techniques (Table 1) have been designed during the past four decades to manage pancreatic endocrine and exocrine secretions after the first successful pancreas transplantation (PTx) performed by Kelly and Lillehei [1], including bladder drainage [2, 3], enteric drainage with and without Roux-en-Y [4], ureteral drainage [5], and pancreas duct ligation and injection [6, 7]. Exocrine drainage to the bladder besides being non-invasive, has the advantages of diagnosing pancreatic rejection by measuring amylase in the urine, which is a non-invasive tool. In contrast to patients undergoing PTx with bladder drainage, patients with enteric pancreas drainage do not experience volume depletion, metabolic acidosis and urologic complications as seen with bladder drainage [8]. Unfortunately, a non-invasive access such as urinary amylase measurement as a marker to detect acute rejection is lost in transplanting pancreas with enteric drainage [2]. Currently, diagnosis of acute rejection in PTx with enteric drainage relies on percutaneous, laparoscopic, or open surgical biopsy [9]. Previously, our group modified Shokouh-Amiri and Gaber’s technique of porto-enteric (P-E) PTx [10, 11] with Roux-en-Y venting jejunostomy [12] as an approach to monitor pancreatic rejection by donor duodenal/pancreatic biopsy and ostomy fluid amylase measurement [13]. In October, 2007 we performed the first simultaneous kidney-pancreas transplant with portal endocrine and gastric exocrine drainage.

**Table 1:** Historical background of pancreatic transplantation

Author(s)	Year	Technique
Kelly and Lillehei	1967	1^st^ pancreatic transplant
Groth	1984	Segmental pancreatic transplantation with enteric exocrine drainage
Calne RY	1984	1^st^ segmental pancreatic transplant with gastric/splenic drainage
Sollinger	1984	Clinical and experimental experience with pancreatico-cystotomy for exocrine pancreatic drainage in pancreas transplantation
Gil-Vemet J	1985	Segmental pancreatic transplant with pylostomy/portal drainage
Nghiem	1986	Duodeno-cystostomy for exocrine drainage in total pancreatic transplantation
Mühlbacher	1990	Splenic vein E-S portal vein and bladder drainage
Shokouh-Amiri and Gaber	1992	Whole pancreatic transplant with enteric/portal Drainage
Rosenlof	1992	Simultaneous bilateral kidney/pancreas transplant
Fridell	2004	Ipsilateral placement of simultaneous pancreas and kidney allografts
Boggi	2004	Retroperitoneal pancreas transplantation with portal-enteric drainage
Dafoe & Ratner	2005	Pancreas/renal composite graft with renal vessel anastomosed to splenic donor vessel
Zibari and Shokouh-Amiri	2007	Portal venous and gastro-jejunostomy drainage
Kornbery	2009	Duodeno-duodenatomy

We have reported earlier our initial experience in the first 10 patients who received PTx, with this technique and demonstrated that this technique is safe and feasible [14]. Since then we have used this technique routinely in all of our patients (n=30) who needed SKP, PTA or PAK. This includes one patient from Labbafinejad Hospital in Tehran, Iran who received a kidney pancreas transplant with P-G technique in 2008 during our NIKI (Network of Iranians for Knowledge and Innovation) trip to Iran in collaboration with Professor Nasser Simforoosh. The purpose of this paper is to report the early and midterm results of PTx in our patients in whom we used this technique.

## PATIENTS AND METHODS

This is an IRB approved retrospective study of 30 patients who underwent PTx (SKP, PTA and PAK) with porto-gastric technique from October 2007 to December 2009 with V-J technique. Demographic data of donors and recipients as well as number of HLA antigen match and CMV status in these patients are shown in Table 2.

**Table 2:** Demographic data of patients (recipient and donor) who received pancreas transplant with porto-gastric technique

Demographic Data	n=30
Mean recipient age (range) (yrs)	40 (16–59)
Gender %	
Male Female	55% 45%
Race	
Caucasian African American Hispanic	20 10 0
Mean duration of diabetes (range) (yrs)	25.5 (6–38)
Simultaneous kidney/pancreas	24
Pancreas transplant alone	4
ancreas after kidney transplant	2
Mean donor age (range) (yrs)	21 (13–38)
Positive CMV status	
Donor Recipient	75% 50%
Human leukocyte antigen (HLA) match	1.3 (0–4)

**Figure 1:**
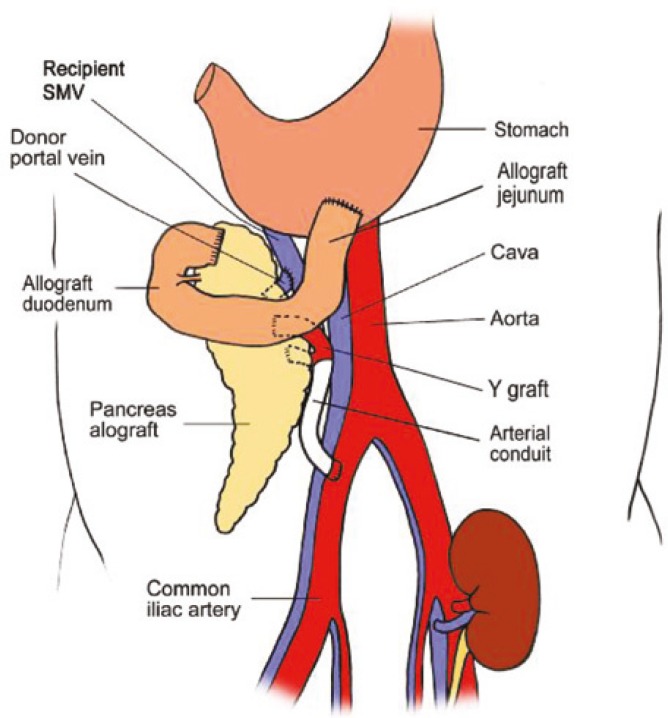
Schematic drawing of the portal-gastric technique for pancreas transplant.with leak compared to those without leak

Surgical procedure:

The standard technique of pancreas and kidney organ procurement for transplantation was slightly modified by recovering a few cm (10–12 cm) more of proximal jejunum with donor pancreas for these patients (Fig 1).

Additionally, we recover long donor iliac arterial Y-conduits and if this is not adequate, we can recover right carotid artery through mediastinal incision. This carotid arterial conduit will be anastomosed end-to-side to recipient right common iliac artery prior to kidney/pancreas transplant (Fig 2A). This donor arterial conduit will be brought through mesentery of distal ileum toward superior mesenteric vein. While one surgeon does donor pancreas bench work in back table the other surgeon performs standard kidney transplant in retroperitoneum at left iliac fosse via the same midline approach. Then, donor pancreas is transplanted via anastomising end of pancreatic allograft portal vein to side of recipient superior mesenteric vein; this is immediately followed by end-to-end anastomosis of reconstructed donor iliac arterial Y-graft of pancreas to previously placed donor arterial conduit anastomosed to right common iliac artery (Fig 2B). At this stage the organ is reperfused. This is followed by donor splenectomy utilizing vascular stapler. The procedure will be completed by anastomising the fully vascularized end of donor jejunum (10–12 cm longer than the standard C-loop of duodenum of pancreas allograft used in P-E PTx technique) to anterior aspect of the stomach close to greater curvature in an anti-colic fashion with a two layer hand sewn anastomosis (Fig 3).

**Figure 2A:**
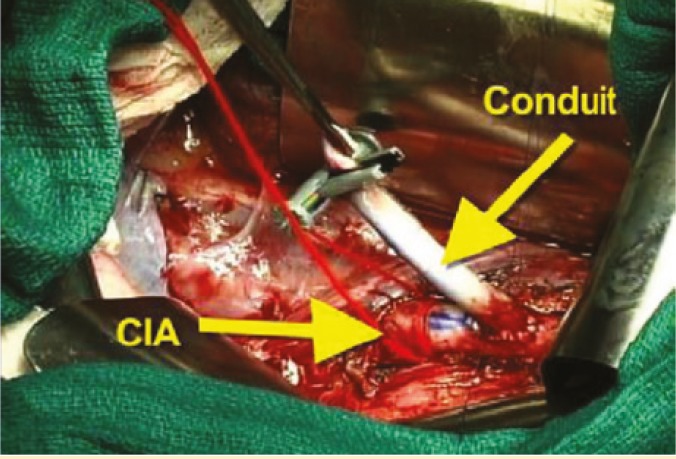
Construction of arterial conduit based on common iliac artery (CIA) for pancreas transplant

**Figure 2B:**
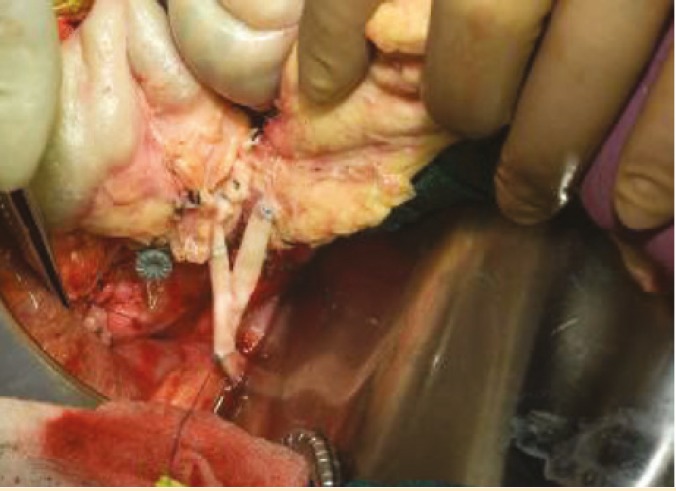
Y-graft anastomosis of pancreas allograft to arterial conduit

**Figure 3:**
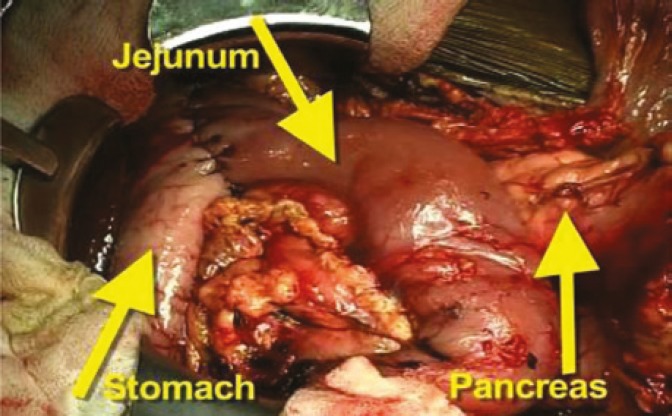
Photograph of completed pancreas allograft implanted with portal-gastric technique

Post-operative management: 

All patients were anticoagulated with systemic heparin in the operating room. Aspirin was given as soon as the patient tolerated oral intake. All patients were treated with antibody induction (thymoglobulin, 1 mg/kg/day for 4–5 days), steroid with rapid tapered dose, tacrolimus, and mycophenolate mofetil. All patients were also treated prophylactically with anti-*Pneumocystis carinii*, antifungal as well as anti-CMV medications for three months. Gastrojejunostomy of pancreas allograft were used for assessment of allograft dysfunction by endoscopic biopsy of allograft duodenum. Patients underwent endoscopic biopsy of donor duodenum/pancreas when acute rejection, CMV disease, or GI bleeding was suspected in addition to obtaining Doppler ultrasound of blood vessels of transplanted pancreas. Perioperative data of patients and graft survival as well as any long-term complications were collected in these patients.

## RESULTS

This new technique of portal-endocrine and gastric-exocrine drainage in PTx has been performed in 30 patients from October 2007 to December 2009 with follow up of 12–38 months. Table 2 shows demographic data of donors and recipients. Tables 3 and 4 show perioperative and outcome data of these patients. All patients achieved insulin free euglycemia and good renal function. 

**Table 3:** Peri-operative and outcome data of patients who received pancreas transplant with porto-gastric technique

Peri-operative and outcome data	n=30
Mean operating time (range) (min)	337 (279–450)
Mean cold ischemia time (range) (min)	587 (240–1194)
Mean intraoperative blood transfusion (range)	1.19 (0–3)
Mean post-operative blood transfusion (range) (units)	1.2 (0–11)
Mean time to feeding (range) (d)	4.8 (3–8)
Mean length of stay (range) (d)	10.5 (6–21)
Major complications	
Thrombosis Bleeding Abscess Hernia	2 1 1 0
Number of endoscopy during follow-up	28 (0–5)
CMV	7
Rejection	4
Endoscopy	0
Mean follow-up time (range) (m)	23 (10–36)

**Table 4:** Patient and graft survival (n=30)

Survival in year	Graft	Patient
1	85%	94%
2	81%	90%
3	76%	90%

Two of our patients suffered vessel thrombosis; one patient developed an anastomotic leak. Three patients died with functioning pancreas and kidney allograft at 17, 28 and 385 days post-transplantation.

**Figure 4:**
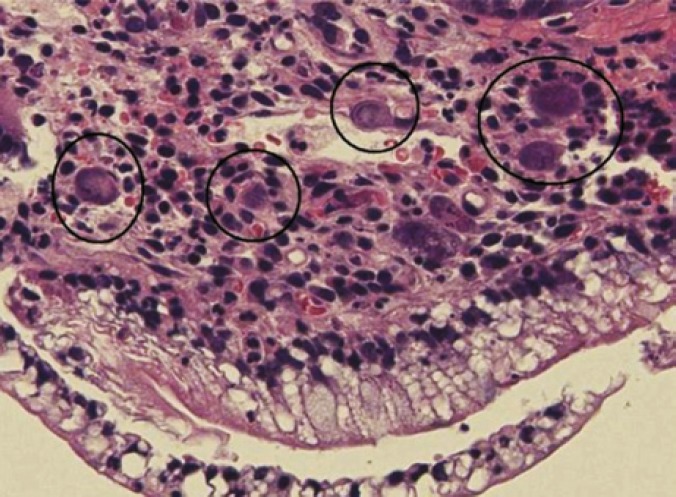
Microscopic picture of CMV inclusion bodies in donor duodenal biopsy

Endoscopy and biopsy of donor duodenum has been performed 28 times in these 30 patients. Seven patients were found to have CMV (Fig 4) and four patients had rejection (Figs 5A, B, C and D). These were treated accordingly during the follow-up. No extra procedure-related complications or readmissions in these patients have been recorded.

**Figure 5A:**
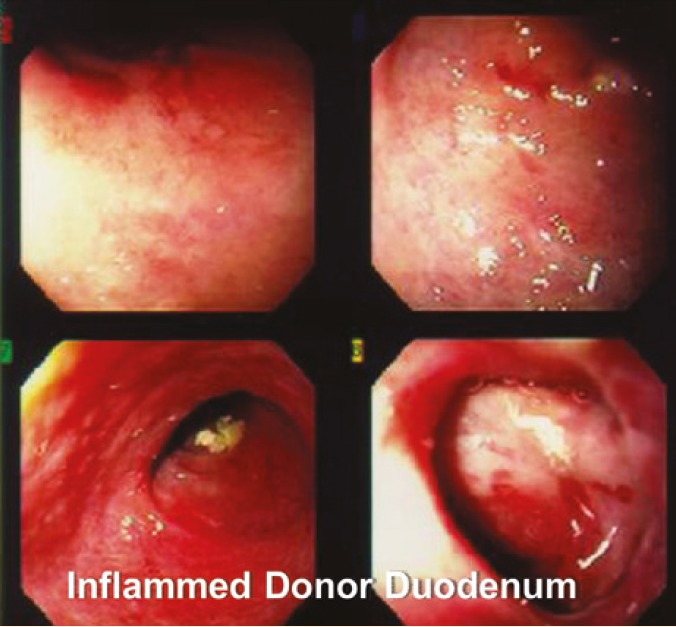
Endoscopic picture of donor duodenum with acute rejection

**Figure 5B:**
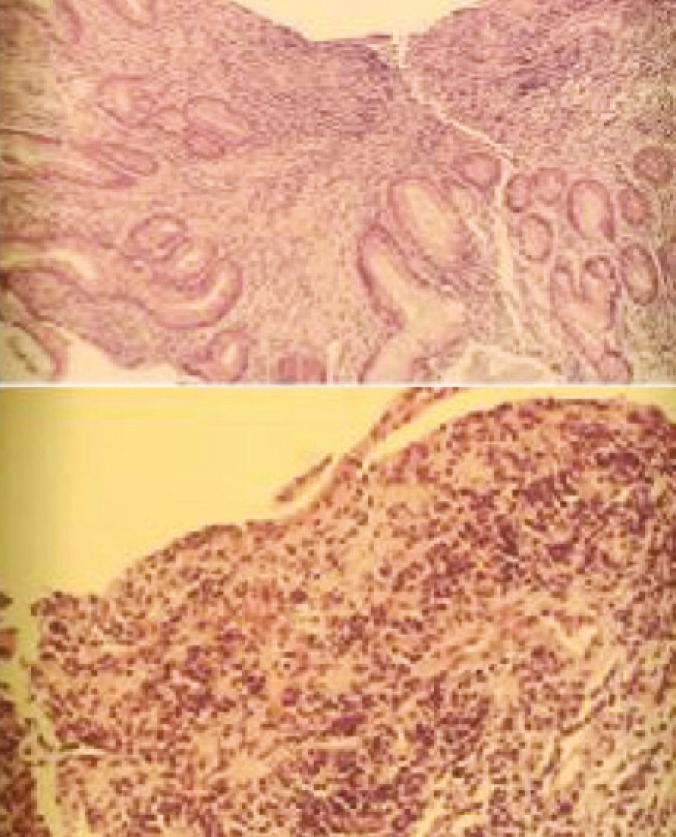
Microscopic picture of donor duodenal biopsy with acute rejection

**Figure 5C:**
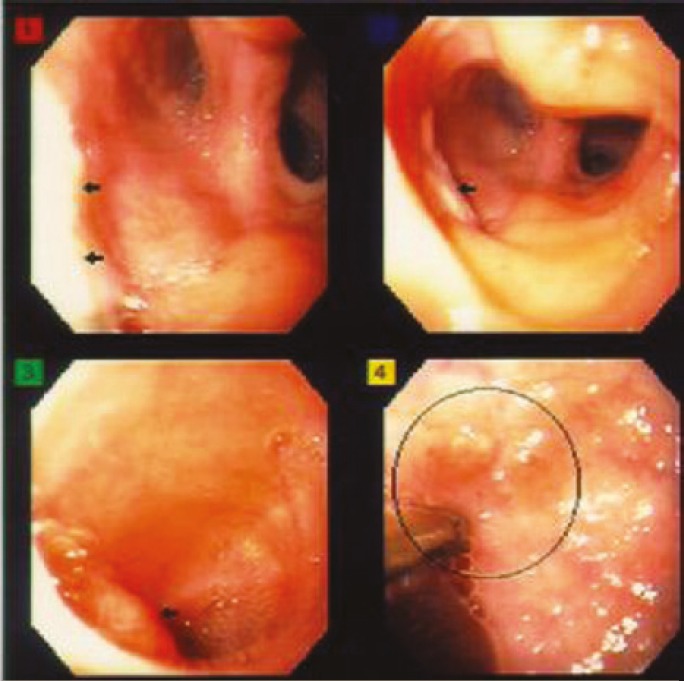
Endoscopic picture of donor duodenum after treatment for acute rejection

**Figure 5D:**
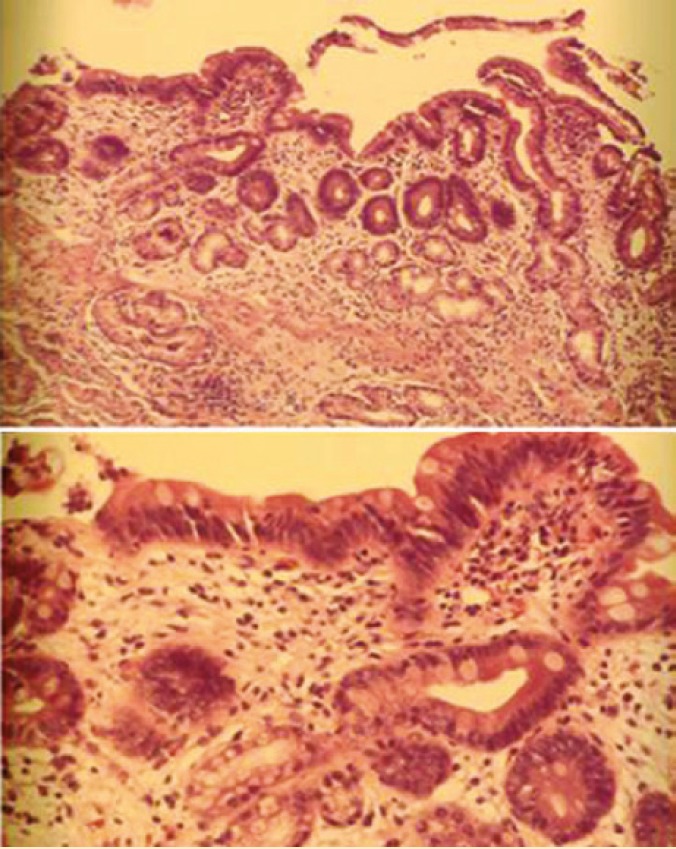
Microscopic picture of donor duodenal biopsy after treatment for acute rejection

Thirty patients received PTx with porto-gastric. Table 2 shows demographic data (age, sex, race, duration of DM, percentage of SKP, PTA, and PAK) in these patients. Donor demographic is also shown in Table 2, length of hospital stay, number of intra-operative blood transfusions as well as overall blood transfusions during post-operative time (1.2 units). Cold ischemia time and OR time is shown in Table 3; Table 4 shows graft and patient survival.

## DISCUSSION

Since the first successful PTx reported by Kelley and Lillehei at the University of Minnesota in 1967 [1], there have been many surgical techniques of pancreas transplant reported by several authors. The initial attempt of a more physiologic approach to PTx by applying segmental PTx with portal and gastric drainage was undertaken by Professor Sir Roy Calne in 1984 [15].

Subsequently, other authors performed pylostomy and portal drainage [5] or enteric drainage and used mesenteric vessels to improve the technique of PTx [16]. In 1990, Muhlbacher, *et al*, described a technique of end-to-side anastomosis between distal splenic vein of the donor and recipient portal vein with bladder drainage [17].

Rosenlof, *et al*, applied Calne’s original technique to whole PTx using an end-to-side anastomosis of donor portal vein and the recipient splenic vein coupled with enteric drainage [18].

Whole organ PTx using the P-E technique was first described by Shokouh-Amiri and Gaber from the University of Tennessee in 1992. This new technique utilized the tributary of SMV to re-establish portal venous drainage [10].

At the present time, the number of pancreas transplant procedures performed worldwide with enteric drainage has increased to 80% of all PTx cases. However, the portal drainage only accounts for 15%–20% of cases [19].

Since Shokouh-Amiri/Gaber popularized the technique of P-E PTx, a number of surgical modifications to PTx techniques have been reported in recent years including P-E drainage with Roux-en-Y venting jejunostomy [12], pediatric enblock duel kidney PTx [20], retroperitoneal pancreas transplant with P-E drainage [21], ipsilateral placement of simultaneous pancreas and kidney allograft [22] laparoscopic simultaneous nephrectomy and distal pancreatectomy [23] and unusual vascular graft [24-27], simultaneous bilateral kidney and PTx to reduce operating time [28] and PTx with duodeno-duodenostomy [29].

The modern surgical era of PTx began with the systemic-bladder drainage technique that reliably achieved an insulin-independent euglycemic state resulting in the successful treatment of DM [2, 3]. The trade off for normal glucose homeostasis is the operative risks of the PTx procedure and the need for lifetime immunosuppression treatment. With advances in clinical immunosuppression [30-32], organ preservation, antimicrobial prophylaxis, and refinements in diagnostic technology and surgical techniques, the success rate for PTx has steadily increased [33, 34].

Although bladder drainage [2, 3] revolutionized the safety of PTx, it was associated with many complications including dehydration, metabolic acidosis and urologic complications, requiring conversion to enteric drainage [15%–26 %] within three years from transplant procedure [35, 36].

The patients who underwent P-E PTx do not have the volume depletion, metabolic acidosis and urologic complications experienced by the patients who underwent bladder-systemic PTx. Though, lack of a non-invasive access to diagnostic markers, such as urinary amylase to detect acute rejection has remained the major disadvantage to long-term follow-up of such patients. Currently, diagnosis of acute rejection in PTx with enteric drainage relies on ultrasound guided percutaneous biopsy [9], laparoscopic or open surgical biopsies. Previously, our group has described a technique of PTx with Roux-en-Y venting jejunostomy [12] as an approach to monitor for rejection, prevent and diagnose anastomotic leak and to diagnose and manage donor duodenal and anastomotic bleed. Unfortunately, in addition of managing and dealing with venting jejunostomy for a period of 6–12 months, there was also a need for subsequent ostomy take down [13] and this lead to loss of access to donor duodenum.

In the fall of 2007, we modified our technique of P-E drainage with Roux-en-Y venting jejunostomy to portal-endocrine and gastric-exocrine drainage. The patients on the waiting list for PTx (SKP, PTA and PAK) were informed of the new procedure and consent was obtained. The enteric-gastric drainage does allow us indefinite easy endoscopic access to both donor duodenum and pancreas allograft. Both pancreas and donor duodenum can be safely biopsied endoscopically to monitor for evidence of acute rejection and CMV duodenitis. Additionally, donor duodenal bleeding as well as anastomotic bleeding can be diagnosed and managed endoscopically in this technique, and P-G patients keep this route of access indefinitely. Endoscopy of allograft duodenum was performed in 28 patients for diagnosis of rejection and CMV disease; the patients were then treated accordingly. Endoscopy has been an easy procedure and no complications have occurred due to this procedure.

In the P-G technique, 10–12 extra cm of proximal jejunum is being used to minimize tension on gastrojejunal anastomosis (Fig 1). This part of the bowel is very well vascularized and poses no danger of ischemia to the graft as the pancreas allograft is procured with SMA and all branches to the head of the pancreas and first part of jejunum is well preserved. There has been one leak from this anastomosis so far, which is not different from other techniques. In this patient, the anastomosis was revised**. **In another patient who required transplant pancreatectomy 28 months after PTx, the jejuno-gastrostomy anastomosis was taken down and the stomach defect repaired. If leak happens, besides revising jejuno-gastrostomy anastomosis, the anastomosis can be taken down and anastomose end of the C-loop of duodenum of allograft to bowel safely. We have not yet observed any case of GVHD or increased rejection rate due to this 10–12 extra cm of bowel.

Regarding the fear that this anastomosis and jejunum is exposed to acidity of the stomach and increased incidence of ulcer formation, only one ulcer has been detected so far. The reason for this low incidence can be due to the neutralizing effect of alkaline secretions of the pancreas and absence of bile in the secretions, since the duodenal loop is a conduit only for pancreas allograft secretion which makes it different from regular gastro-jejunostomy that is used in general surgery with its increased incidence of marginal ulcer. The technique of P-E PTx has been in use since 1990 and several hundred patients have been transplanted with this technique safely. Furthermore, in author’s opinion this technique is even easier than the systemic enteric or bladder drainage techniques. Placement of an arterial conduit first, and then anastomosing the reconstructed arterial supply of pancreas allograft to the conduit makes the technique much easier compared to the original technique that brought the reconstructed arterial supply of pancreas allograft to right common iliac artery after portal venous anastomosis. This prevents the potential twisting of the artery and also prevents pressure on the allograft during arterial anastomosis. This is the observation and experience of the co-author of this technique, Hosein Shokouh-Amiri, who have designed the P-E PTx and performed numerous PTx with P-E technique, as well as systemic-enteric/systemic-bladder techniques. In more than 300 P-E PTx performed by the authors, a total of 4–5 PV thrombosis have been observed. These have been managed safely by graft pancreatectomy, portal vein embolectomy and repair of SMV stump at the site of the anastomosis. In 2000, we reported our experience in reusing the same SMV in P-E PTx successfully in five pancreas re-transplant patients without any difficulty [37]. Since then, we have reused the same SMV in few more patients requiring re-transplant. The requirement for this is that during the transplant pancreatectomy one should leave behind 5–10 mm stump of allograft portal vein, so the recipient SMV does not get narrow.

Before applying to clinical setting, we did design an animal model of P-E PTx with duodeno-duodenostomy for exocrine drainage [38], but due to the theoretical fear of leak from this anastomosis which puts the patient in a much higher risk, especially in an immunosuppressed patient compared to the leak from gastro-jejunostomy or jejuno-jejunostomy, which can be handled much easier by taking down the anastomosis, we have never used the duodeno-duodenostomy and do not advise the use of it, even though Kornberg, *et al*, have used it successfully in their patients [29]. The initial use of stomach as exocrine drainage site has been in the form of segmental PTx with invagination of pancreatic stump into the stomach [15] and the reason for the lack of interest in pursuing this technique has possibly been due to the overall complexity of the technique using portal vein to splenic vein anastomosis which lies in a much more difficult location for a venous anastomosis. The type of drainage of allograft exocrine to stomach in the form we are presenting is quiet new and unique to this technique. This facilitates lifelong access to the allograft if it became necessary, without losing fluids or electrolytes (which have been the main problem with bladder drainage). One allograft pancreatitis without long-term sequela, but no protein loosing enteropathies has occurred so far in this limited number of patients. Gastro-jejunostomy can be performed end-to-side or side-to-side based on the surgeon’s preference.

Our preliminary results of PTx with gastric-exocrine and portal-endocrine drainage show that this is a safe technique with minimal complications, as well as good patients and allograft survival. The added advantage of permanent and easy access to donor duodenum and pancreas allograft endoscopically is unique to this novel technique of PTx [39]. This gastric-exocrine pancreatic drainage should be even more useful in settings of solitary PTx and with pancreas after kidney transplantation where other markers for diagnosing rejection such as serum creatinine or urine creatinine will not be applicable to pancreas allograft functions. Longer follow-up and larger multi-center studies are needed to reconfirm our results.
